# Supplementary material for: Changes in neurotensin signalling drive hedonic devaluation in obesity
Source: Nature. 2025 Mar 26;641(8065):1238–47. doi: 10.1038/s41586-025-08748-y (PMC12119351; doi:10.1038/s41586-025-08748-y)
Supplement: Supplementary file 1 — Reporting Summary [file 41586_2025_8748_MOESM1_ESM.pdf]

nature portfolio

Corresponding author(s): Stephan Lammel

Last updated by author(s): Jan 22, 2025

Reporting Summary

Nature Portfolio wishes to improve the reproducibility of the work that we publish. This form provides structure for consistency and transparency in reporting. For further information on Nature Portfolio policies, see our Editorial Policies and the Editorial Policy Checklist.

Statistics

For all statistical analyses, confirm that the following items are present in the figure legend, table legend, main text, or Methods section.

n/a	Confirmed	
☐	☒	The exact sample size (*n*) for each experimental group/condition, given as a discrete number and unit of measurement
☐	☒	A statement on whether measurements were taken from distinct samples or whether the same sample was measured repeatedly
☐	☒	The statistical test(s) used AND whether they are one- or two-sided<br>*Only common tests should be described solely by name; describe more complex techniques in the Methods section.*
☐	☒	A description of all covariates tested
☐	☒	A description of any assumptions or corrections, such as tests of normality and adjustment for multiple comparisons
☐	☒	A full description of the statistical parameters including central tendency (e.g. means) or other basic estimates (e.g. regression coefficient) AND variation (e.g. standard deviation) or associated estimates of uncertainty (e.g. confidence intervals)
☐	☒	For null hypothesis testing, the test statistic (e.g. *F*, *t*, *r*) with confidence intervals, effect sizes, degrees of freedom and *P* value noted<br>*Give P values as exact values whenever suitable.*
☒	☐	For Bayesian analysis, information on the choice of priors and Markov chain Monte Carlo settings
☒	☐	For hierarchical and complex designs, identification of the appropriate level for tests and full reporting of outcomes
☐	☒	Estimates of effect sizes (e.g. Cohen's *d*, Pearson's *r*), indicating how they were calculated

Our web collection on statistics for biologists contains articles on many of the points above.

Software and code

Policy information about availability of computer code

Data collection	PClamp 10.5 (Molecular Devices) was used for acquisition of brain slice electrophysiology data. Zen Software 2.3 (Zeiss) was used for acquiring confocal and epifluorescence images of brain slices. For validation and spectral analysis experiments of ntsLight1.1 and 2.0 cultured neurons were imaged with a Leica Stellaris 8 Confocal. For imaging of ntsLight1.1 in brain slices, a custom-built, open source macroscope (https://github.com/Llamero/DIY_Epifluorescence_Macroscope) fitted with high power LEDs and a Teledyne Kinetix sCMOS camera. Imaging of neuronal cultures was performed using MetaXpress software (Version 6.6.3.55) on the ImageXpress MicroConfocal system. Neural signals were recording used using a Digital Lynx 4SX system. ntsLight2.0 signals were recorded using a MATLAB-based custom fiber photometry system https://github.com/handejong/Fipster. For in vivo electrophysiology experiments, neural signals were recorded using a Digital Lynx 4SX system. Laser output was controlled using a Master-8 pulse stimulator (A.M.P.I.). Spikes were sorted offline using SpikeSort3D 2.5.4 (Neuralynx) software. Video-based offline tracking was performed via DeepLabCut.py (Version 2.0.7). Biobserve (Version 3.0.1.442) video tracking system was used for the real-time place preference test. In RNAseq experiments, cells were pooled and sequenced using the Illumina NovaSeq 6000 with 150 bp paired-end reads. After sequencing, raw reads were de-multiplexed using Illumina bc12fastq (Version 2.20), and pseudo-aligned to the Ensembl GRCm38.95 reference transcriptome and normalized using kallisto (Version 0.45.1).
Data analysis	Comparative statistical tests, correlation, and regression were performed in GraphPad Prism Versions 9 (Version 9.5.1) and 10 (Version 10.3.1). Gene expression analysis for RNAseq was performed using Python (Version 3.6.7), R (Version 3.5.1) and edgeR (Version 3.24.3). MouseActivity5.m was used to analyze open-field behavior (https://github.com/HanLab-OSU/MouseActivity/blob/master/MouseActivity5.m). DeepLabCut.py (Version 2.0.7) was used to analyze mouse behavior. For analysis of in vivo electrophysiology data, NeuralynxMatlabImportExport_v6.0.0 MATLAB package was used, which is available at https://neuralynx.fh-co.com/research-software/. Custom MATLAB (Version R2024a), Python (Version 3.6.7) and edgeR (Version 3.24.3) were used for the processing of in vivo electrophysiology data and RNAseq are available at https://github.com/lammellab/. ImageJ (NIH, 64-bit Java 1.8.0_172) was used for analysis of fluorescence and confocal images. For Ligand specificity test in cell cultures, images from Leica Stellaris Confocal were exported and

analyzed using a customized MATLAB (Version R2023b) script available at https://github.com/lintianlab. Brain slice electrophysiology data were analyzed offline using Clampfit (Molecular Devices, Version 10.5).

For manuscripts utilizing custom algorithms or software that are central to the research but not yet described in published literature, software must be made available to editors and reviewers. We strongly encourage code deposition in a community repository (e.g. GitHub). See the Nature Portfolio guidelines for submitting code & software for further information.

Data

Policy information about availability of data

All manuscripts must include a data availability statement. This statement should provide the following information, where applicable:

- Accession codes, unique identifiers, or web links for publicly available datasets
- A description of any restrictions on data availability
- For clinical datasets or third party data, please ensure that the statement adheres to our policy

All source data are available in the accompanying source data files. The RNAseq datasets generated during this study are available at NCBI GEO; GSE287548.

Research involving human participants, their data, or biological material

Policy information about studies with human participants or human data. See also policy information about sex, gender (identity/presentation), and sexual orientation and race, ethnicity and racism.

Reporting on sex and gender	N/A
Reporting on race, ethnicity, or other socially relevant groupings	N/A
Population characteristics	N/A
Recruitment	N/A
Ethics oversight	N/A

Note that full information on the approval of the study protocol must also be provided in the manuscript.

Field-specific reporting

Please select the one below that is the best fit for your research. If you are not sure, read the appropriate sections before making your selection.

☒ Life sciences ☐ Behavioural & social sciences ☐ Ecological, evolutionary & environmental sciences

For a reference copy of the document with all sections, see nature.com/documents/nr-reporting-summary-flat.pdf

Life sciences study design

All studies must disclose on these points even when the disclosure is negative.

Sample size	No statistical methods were used to predetermine sample size, which were based on work in previous publications (Lammel et al., 2008; Neuron; Lammel et al., 2012; Nature; Cerniauskas et al., 2019; Neuron; Yang et al., 2021; Nature Neuroscience; Cardozo Pinto et al., 2019; Nature Communications).
Data exclusions	For recordings of NAcLat-VTA units during behavior (Fig. 1), data was excluded from the statistical analyses in the following cases: For DeepLabCut-based analysis: if either of the 10 behavioral motifs was not detected, it was not included in the total cell count for evaluating proportions of DR, IR and non-responsive in the group analysis. For Piezo-based analysis: a trial was excluded if the sensor was not triggered. For the RNAseq experiment (Fig. 3), genes were not included in the differential gene expression analysis unless they met the following criteria: at least 5 cells with a CPM value above 15 and the average CPM value higher than 4.
Replication	Experiments were designed so that the data is based on at least two independent subjects per group. This applies to the following experiments: establishing HFD mouse model (Fig. 1a-d), in vivo electrophysiology (Fig. 1e-m, ED Fig. 1e-r, ED Fig. 2), optogenetics (Fig. 2, Fig. 4e, i; Fig. 5e; ED Fig. 3c-m, p; ED Fig. 4c-f; ED Fig. 8a-d, g, h), RNAseq (Fig. 3a-j; ED Fig. 5a-f), NTS release (Fig. 3k-p; Fig. 5a-c; ED Fig 6a-g; ED Fig. 7a-z; ED Fig. 10d), brain slice electrophysiology (Fig. 4l, m; ED Fig. 9b, c, e, f, j-q), tracing (ED Fig. 5g, h), in situ hybridization (ED Fig. 5i-m; ED Fig. 9g-i; ED Fig. 10a-c) NTS-OE (Fig. 5f-l, ED Fig. 10e-k), but with the only exception of the data shown in ED Fig. 6d, e, which was obtained from one subject. Several of these experiments were replicated in at least two technical replicates from each of at least two mice and similar results were obtained. For example, establishing the HFD mouse model (Fig. 1a-d), optogenetics (Fig. 2, Fig. 4i) and NTS-OE (Fig. 5f-l, ED Fig. 10e-k). For anatomical experiments, wherever representative examples are shown (Fig. 4g, k; ED Fig. 1a-d; ED Fig. 3a, b, n, q-t; ED Fig. 4a, b; ED Fig. 6h; ED Fig. 7aa, ab; ED Fig. 8e, f, i, j; ED Fig. 9a, d), similar results were obtained in at least two technical replicates from each of at least two mice.
Randomization	Animals were randomly assigned to different diet and virus injection groups from litter mates. For recordings of NAcLat-VTA units during

Randomization	behavior (Fig. 1), the order of jelly and chow exposure was randomized between animals. During optogenetic experiments, the order of mice expressing ChR2 or control fluorophore was randomized for each experimental condition.
Blinding	For quantification of NTS-OE using in situ hybridization (ED Fig. 10a-c), all identified regions of interest were manually sorted by an investigator who was blind to virus expression, diet, and probe mix. For quantification of NTS mRNA expression using in situ hybridization following NTS-KO (Fig. 4 b, c) and manipulation of diets (ED Fig. 5i-m), the images were shuffled, and the experimental conditions were hidden during image processing and pixel intensity evaluation. Blinding was not used in other experiments because the experimental conditions were obvious to the researchers and the analysis was performed objectively and not subjective to human bias or analyses were automated.

Reporting for specific materials, systems and methods

We require information from authors about some types of materials, experimental systems and methods used in many studies. Here, indicate whether each material, system or method listed is relevant to your study. If you are not sure if a list item applies to your research, read the appropriate section before selecting a response.

Materials & experimental systems

n/a	Involved in the study
☐	☒ Antibodies
☐	☒ Eukaryotic cell lines
☒	☐ Palaeontology and archaeology
☐	☒ Animals and other organisms
☒	☐ Clinical data
☒	☐ Dual use research of concern
☒	☐ Plants

Methods

n/a	Involved in the study
☒	☐ ChIP-seq
☒	☐ Flow cytometry
☒	☐ MRI-based neuroimaging

Antibodies

Antibodies used	Primary antibodies: rabbit anti-TH (Millipore, 657012), chicken anti-GFP (Abcam, ab13970), rabbit anti-DS Red (Living Colors, Takara Bio 632496). Secondary antibodies: goat anti-rabbit Alexa Fluor 546 (Life Technologies, A11010), goat anti-chicken Alexa Fluor 488 (Abcam, ab150169), all 1:750).
Validation	All antibodies mentioned above have been extensively validated in mice in each of these previous studies (Lammel et al., 2008; Neuron; Lammel et al., 2012; Nature; Cerniauskas et al., 2019; Neuron, Yang et al., 2021; Nature Neuroscience; Cardozo Pinto, et al., 2019), except rabbit anti-DS red antibody, which was validated by the manufacturer: https://www.takarabio.com/documents/Certificate%20of%20Analysis/632496/632496-101717.pdf

Eukaryotic cell lines

Policy information about cell lines and Sex and Gender in Research

Cell line source(s)	HEK293T (ATCC, CRL-3126, https://www.atcc.org/products/crl-3216). E18 rat hippocampal neuron (BrainBits, https://tissue.transnetyx.com/E18-Rat-Hippocampus_4).
Authentication	These cell lines were not authenticated.
Mycoplasma contamination	These cell lines were not tested for mycoplasma contamination.
Commonly misidentified lines (See ICLAC register)	No commonly misidentified cell lines were used in the study.

Animals and other research organisms

Policy information about studies involving animals; ARRIVE guidelines recommended for reporting animal research, and Sex and Gender in Research

Laboratory animals	The following mouse lines (6-8 weeks old) were used: C57BL/6J mice (Jackson Laboratory, stock number: 000664), NTS-Cre (Jackson Laboratory; stock number: 017525, strain code: Ntstm1(cre)Mgmj), GAD2-Cre (Jackson Laboratory, stock number: 010802, strain code: Gad2tm2(cre)Zjh/J), Ai14 (Jackson Laboratory, stock number: 007914, strain code: B6.Cg-Gt(ROSA)26Sortm14(CAG-tdTomato)Hze/J). NTS-FLOX (Jackson Laboratory, stock number: 036262, strain code: B6;FVB-Ntsem1Evdr/J). Mice were housed on a 12:12 light cycle (lights on at 07:00) and a room temperature of 22-25°C and 55% humidity. All procedures complied with the animal care standards set forth by the National Institutes of Health and were approved by University of California Berkeley's Administrative Panel on Laboratory Animal Care.
Wild animals	The study did not involve wild animals.
Reporting on sex	Sex was not considered as a co-variable. All experiments were performed in mixed cohorts of mice. No between-mice comparisons

Reporting on sex	were made in the study.
Field-collected samples	No field-collected samples were used.
Ethics oversight	All procedures complied with the animal care standards set forth by the National Institutes of Health and were approved by University of California, Berkeley's Administrative Panel on Laboratory Animal Care.

Note that full information on the approval of the study protocol must also be provided in the manuscript.

Plants

Seed stocks	N/A
Novel plant genotypes	N/A
Authentication	N/A
